# Notes from Private Practice

**Published:** 1877-08

**Authors:** 


### NOTES FROM PRIVATE PRACTICE.

*Congenital absence of Uterus and Vagina:*

Simply as a matter of record, I report the following case of congenital absence of the uterus and vagina, which lately came under my observation.

Miss H.—aged 19—nationality, Irish—third child in a family of six children, four of whom were boys. She says there is no deformity or arrest of development, either in any of the other children, or anywhere else among any of her other relatives; that all are perfectly formed and healthy.

She has never menstruated, but about once a month she has some headache and a sense of fullness in the abdomen.

She was first led to believe that "something was wrong" with her about a year ago, from the fact that she "did not have her courses as other girls do." She says she enjoys the society of the opposite sex she "supposes the same as any girl does." Apparently she is a perfect specimen of young womanhood—tall, well developed, robust and active. Examination (afterwards confirmed by Dr. W. H. Byford of this city) showed, the parts well covered with hair; labia majora and nymphæ, as well as clitoris and hymen well developed and natural. Meatus urinarius normal in position. Mammæ large and also well developed, but the most careful and rigid examination *failed to find either vagina or womb.* There is, however, no reason to doubt the existence of the ovaries.

Chicago, June, 1877. A. W. Gray, M. D.

---

*Local anæsthesia.*—In the following communication, I wish to call the attention of the reader to a simple method of producing local anæsthesia in some operations of minor surgery, as in opening an abscess, excision of inverted toe-nails, etc. The contrivance is a very old one, yet it is not so thoroughly appreciated by practitioners, as in my opinion, it ought to be, for it is extremely simple and reliable. The principle upon which the method is based, is the fact, that a mixture of equal parts of ice or snow, and common salt produce a very high refrigerating action of from 0° to 17° C = from 32° F to 0° F (according to Hager's text-book). The way to make use of this physical fact in surgery is as follows:

Take pounded ice or snow - one part (spoonful,)
Common salt - - - one part (spoonful)

Put both on a soft rag (musquito netting); mix it to form a kind of poultice and press it quickly against the skin, where you intend to make an incision. In a very short time the skin freezes and becomes absolutely insensible and bloodless, thus affording the double advantage of avoiding any pain and of preventing the blood from obscuring the field of operation. As to the length of time this state of the skin might be maintained without endangering its vitality, I have made no experments; but I think it may be continued a longer time, than is required for the performance of any minor operation.

As the temperature begins sinking the moment the ice and the salt are brought in contact, all that is necessary for the performance of the operation must be ready before making the mixture. Special care must be taken to have the knife as sharp as possible, for the frozen skin is remarkably tough, and resists the instrument to a high degree.

I may add, that, if the fingers or toes be the parts on which an operation is performed, the ice-salt-poultice seems the best method of applying the refrigerating mixture, because we can cover the finger or the toe all around with ice; but on the other hand, if a larger surface (for instance, the breast in a case of mastitis suppurativa) be the object of operation, we best proceed by putting on the spot, previously selected for incision, a piece of ice as large as a hickory-nut, that is then sprinkled with salt. Almost instantly the skin beneath the ice freezes; then we take away the ice and salt and make the incision. That the size of the ice-salt poultice in the one case, and the size of the piece of ice in the other depends, after all, upon the length of the intended incision, is hardly necessary to be mentioned.

The following two cases may be reported to confirm the above statements:

1. Some time ago I was called to a woman, who received a splinter of wood under the nail of the right thumb, while she was washing the floor. I saw the patient 3 days after the accident; the splinter which had advanced as far as the root of the nail was broken short at the edge of the nail, in consequence of previous attempts at pulling it out. A diffuse swelling, throbbing and burning indicated that inflammation had already set in; the patient was nervous and feverish, and was so excited by fear of the knife, that she would hardly allow me to touch the finger. But being assured she would not feel any pain from the operation, she submitted to it. I applied an ice-salt poultice (as described) close around the nail-phalanx of the thumb. After some 2 or 3 minutes the thumb was frozen and completely white and insensible. Having cut open the nail with a pair of scissors all along the splinter, I removed the latter, with the pus which had begun to form around the foreign body, and finally cleansed and dressed the wound, while not a drop of blood came out and while the patient had not the slightest feeling of what was going on.

2. Some 3 weeks ago, I saw a case of mastitis suppurativa in puerpero. The pus was deep-seated, fever high, and the patient, a young married woman of nervous temperament, excited to the highest degree, by the thought that she would have to be cut. For obvious reasons, I insisted upon opening the abscess at once. As in the first case, I had some difficulty in persuading the frightened patient to follow my directions and not to think of pain. Yet, after all, I was allowed to proceed. I put a piece of ice as large as a hickory-nut on the spot, which seemed the most suitable for incision, and sprinkled the ice with common salt. Almost instantly the skin, in the circumference of a quarter of a dollar, became white, and having removed the ice, I plunged the knife through the frozen skin, into the abscess. The patient who lay there shivering from fear, did not feel the incision at all, and was greatly surprised when she opened her eyes, by seeing the pus flow out.

DR. H. BANGA, Chicago.

